# Supplementary figures and images for: Assessing the potential role of copper and cobalt in stimulating angiogenesis for tissue regeneration
Source: PLoS One. 2021 Oct 27;16(10):e0259125. doi: 10.1371/journal.pone.0259125 (PMC8550415; doi:10.1371/journal.pone.0259125)

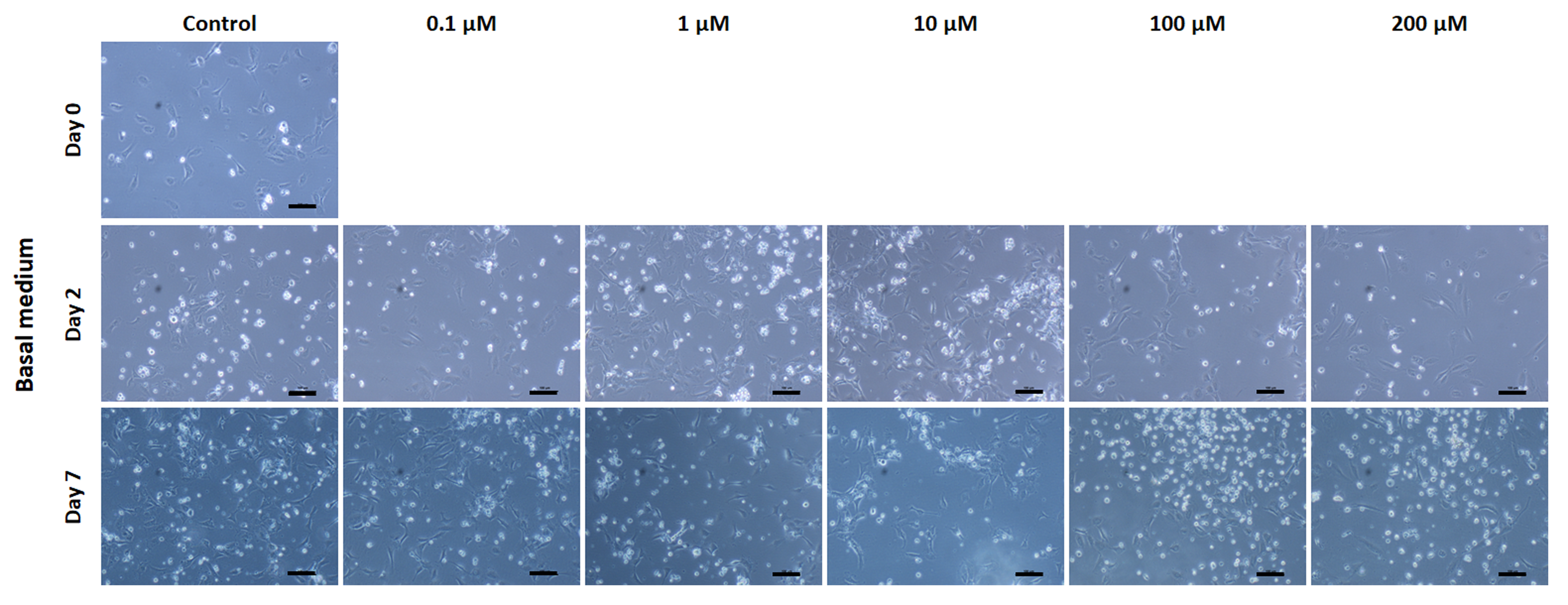

Supplement: S1 Fig — (TIFF) [file pone.0259125.s001.tiff]

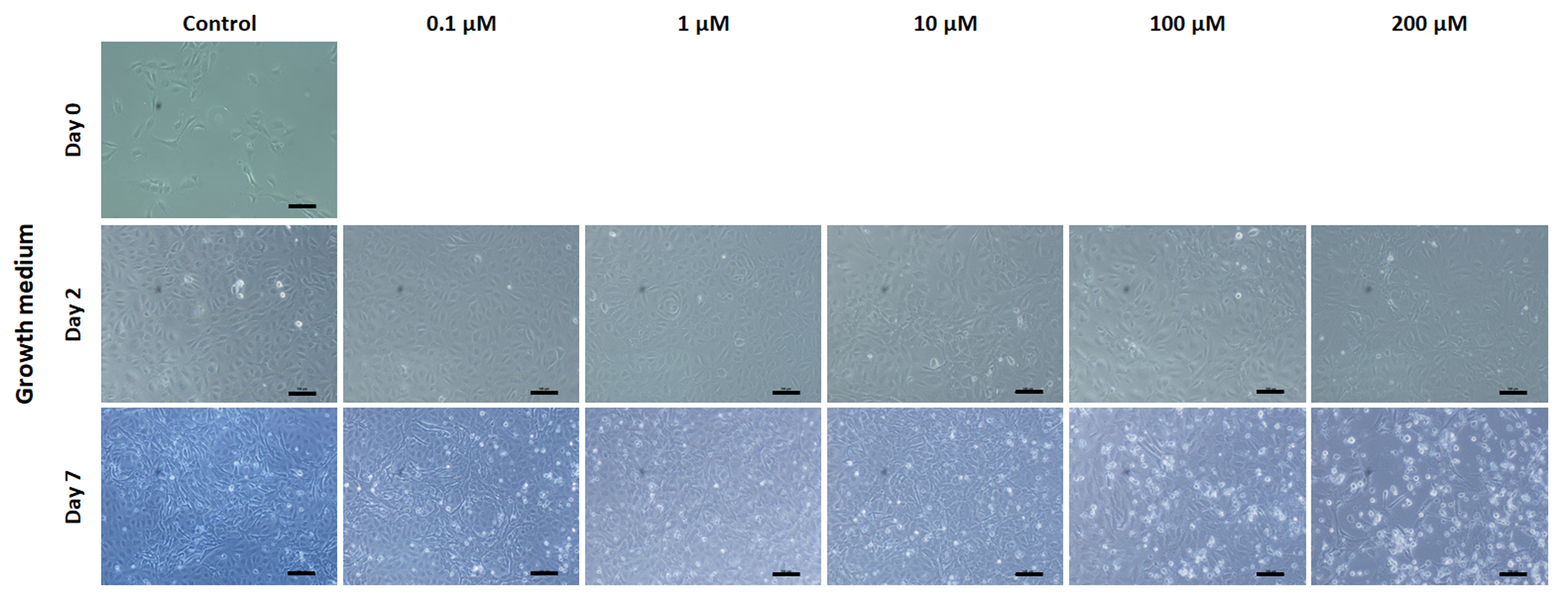

Supplement: S2 Fig — (TIFF) [file pone.0259125.s002.tiff]

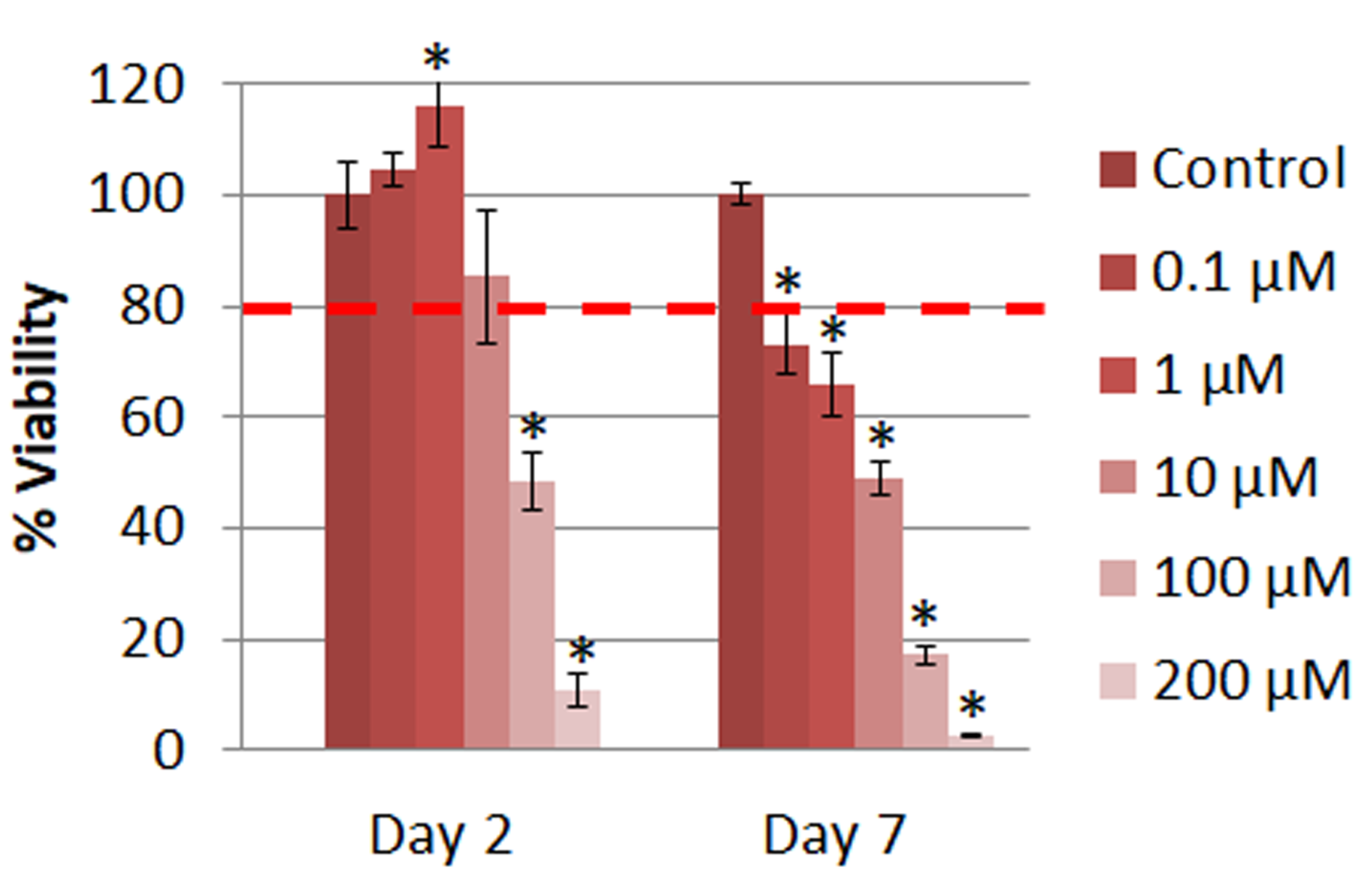

Supplement: S3 Fig — Viability of HUVEC cultured with basal medium supplemented with Cu2+. Statistically significant differences were represented with * (p<0.05). (TIFF) [file pone.0259125.s003.tiff]

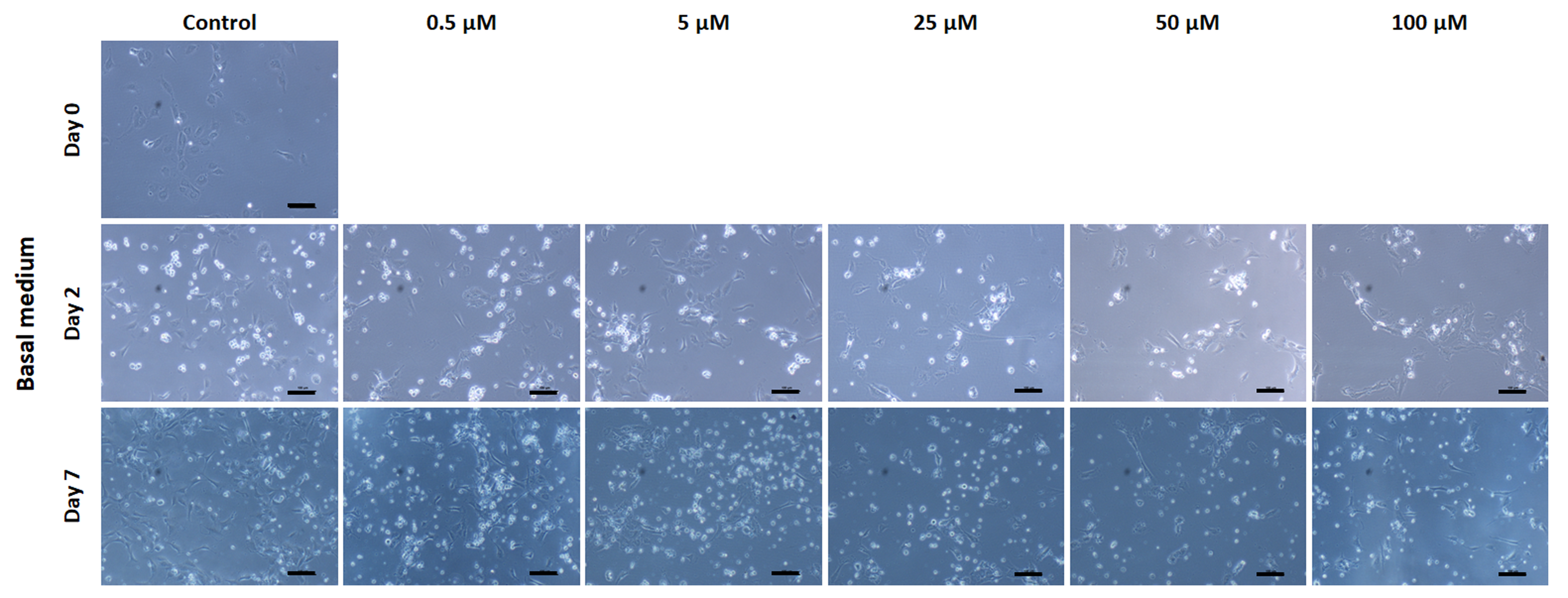

Supplement: S4 Fig — (TIFF) [file pone.0259125.s004.tiff]

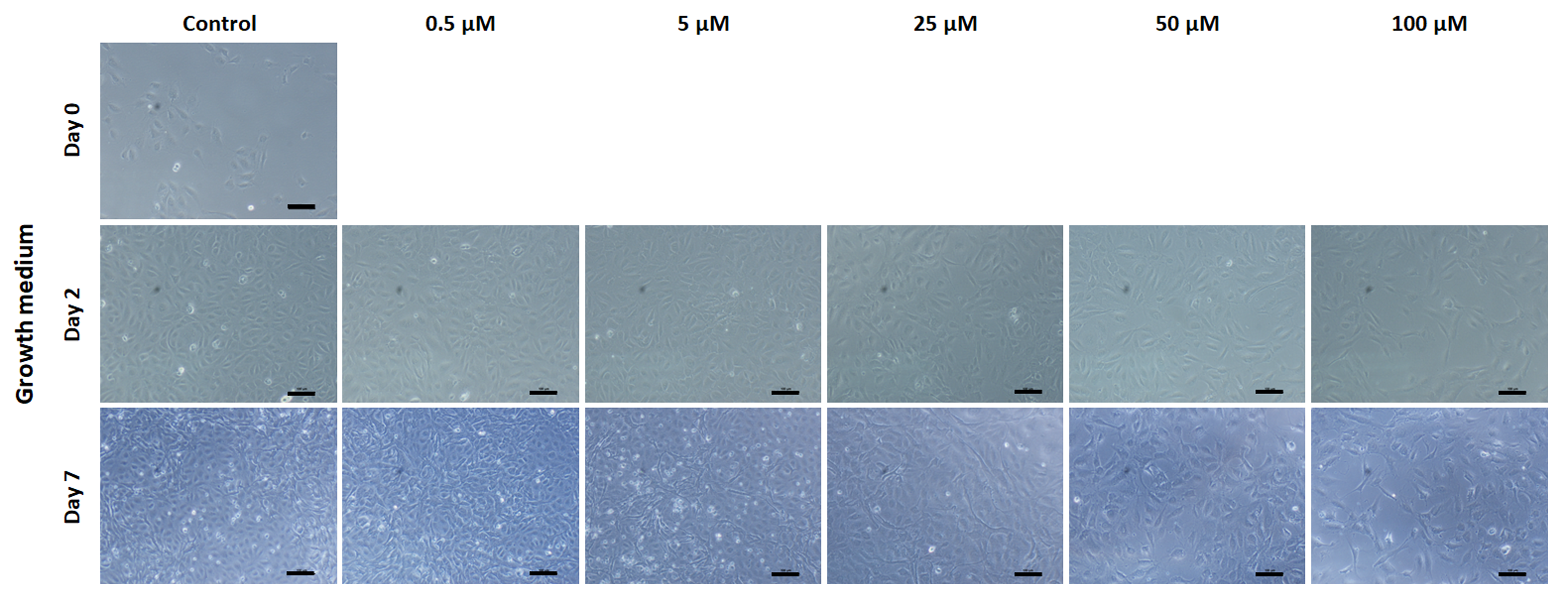

Supplement: S5 Fig — (TIFF) [file pone.0259125.s005.tiff]

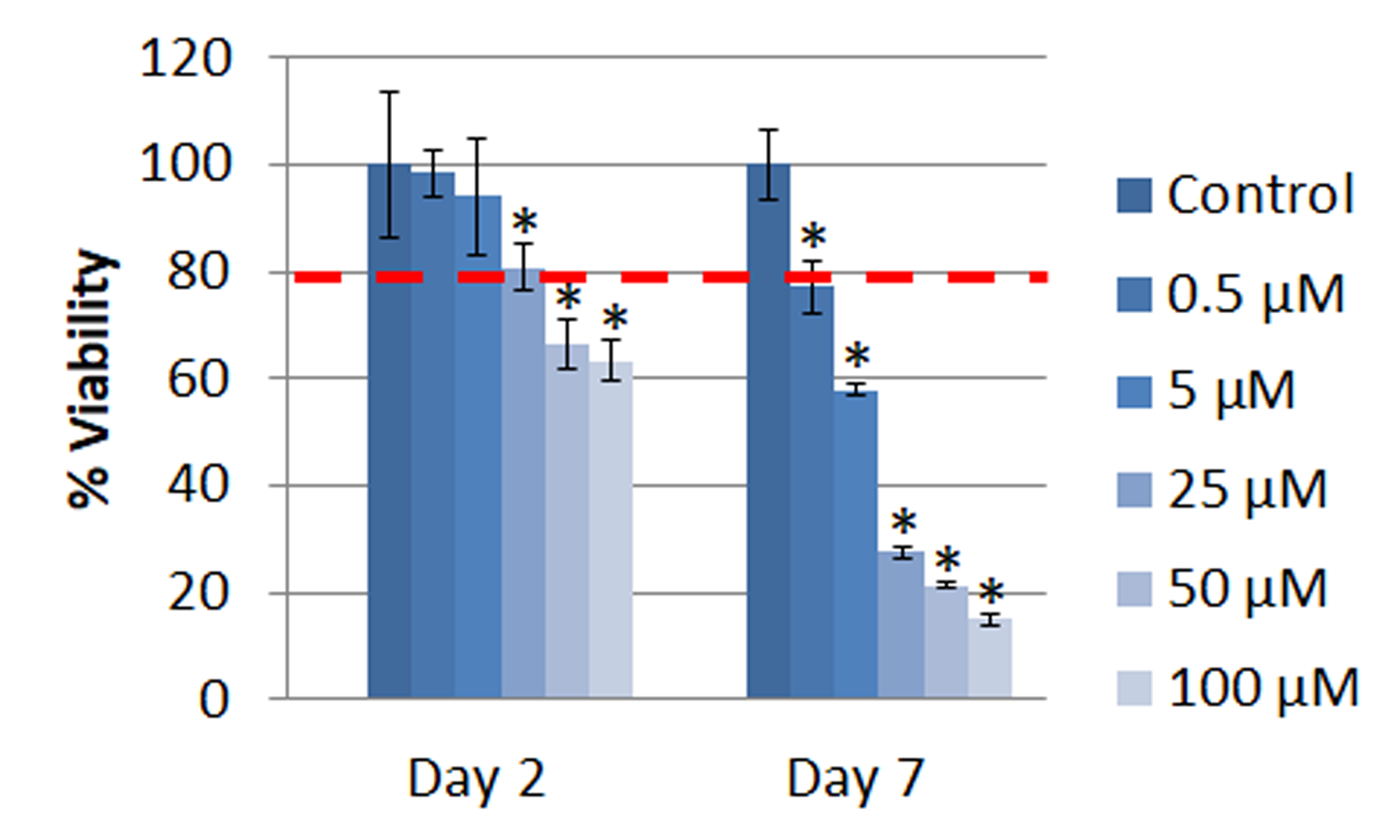

Supplement: S6 Fig — Viability of HUVEC cultured with basal medium supplemented with Co2+. Statistically significant differences were represented with * (p<0.05). (TIF) [file pone.0259125.s006.tif]

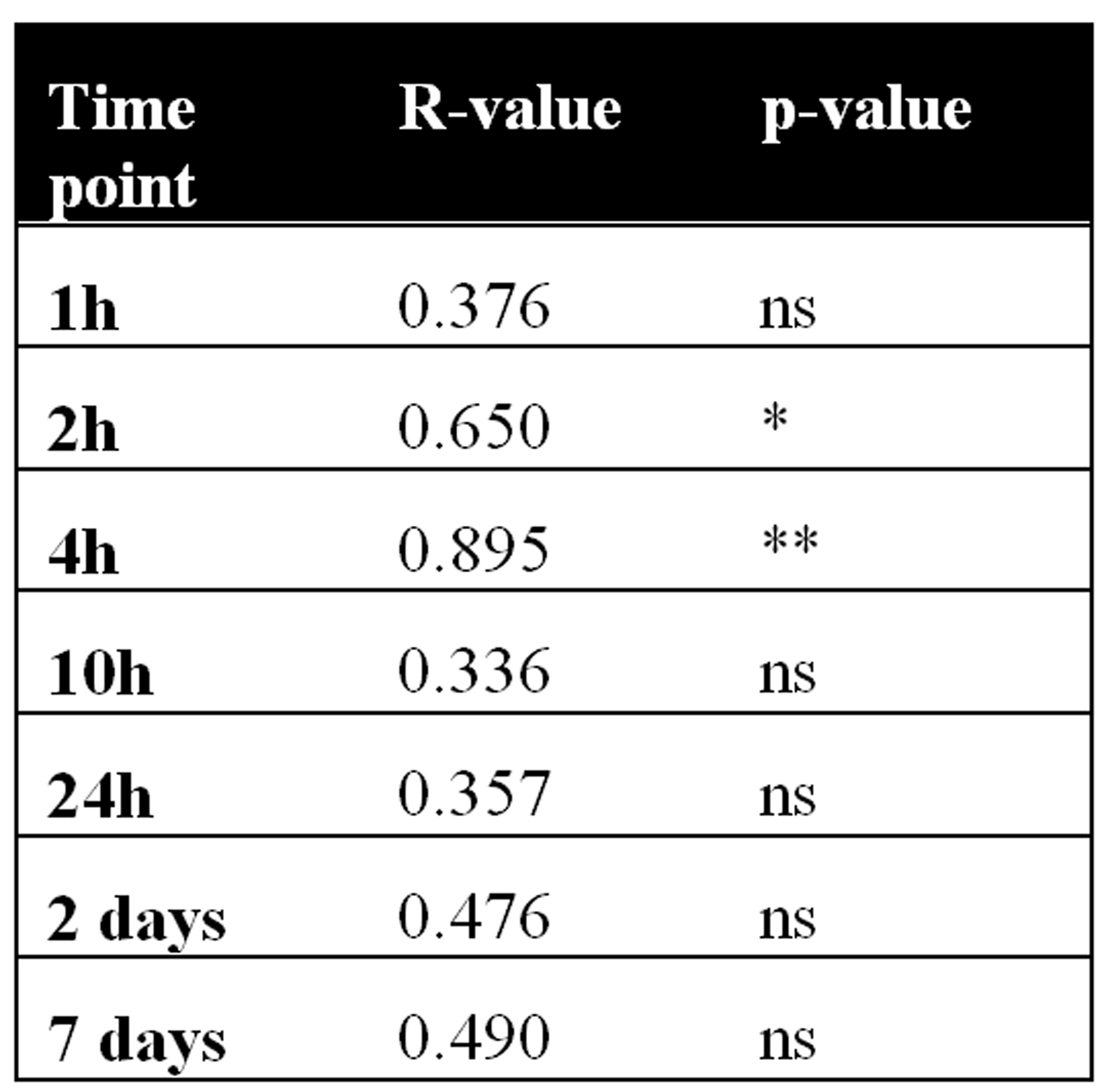

Supplement: S1 Table — Symbols: ns = non-significant (p>0.05); * = p<0.05; ** = p<0.01. (TIF) [file pone.0259125.s007.tif]

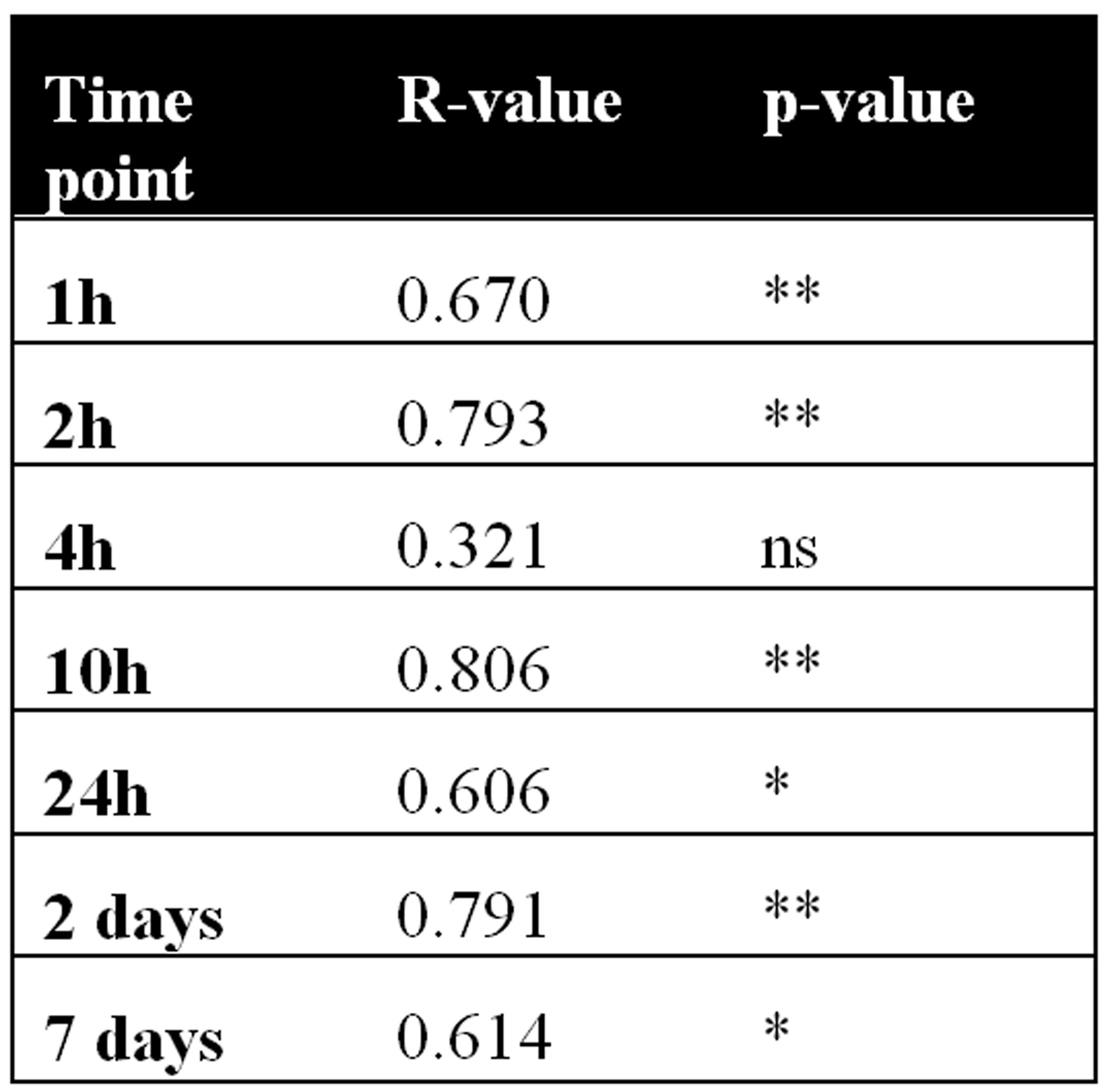

Supplement: S2 Table — Symbols: ns = non-significant (p>0.05); * = p<0.05; ** = p<0.01. (TIF) [file pone.0259125.s008.tif]
